# Leaf morphology in Cowpea [*Vigna unguiculata* (L.) Walp]: QTL analysis, physical mapping and identifying a candidate gene using synteny with model legume species

**DOI:** 10.1186/1471-2164-13-234

**Published:** 2012-06-12

**Authors:** Marti Pottorff, Jeffrey D Ehlers, Christian Fatokun, Philip A Roberts, Timothy J Close

**Affiliations:** 1 Department of Botany & Plant Sciences, University of California Riverside, Riverside, CA, USA; 2 Bill & Melinda Gates Foundation, Seattle, WA, USA; 3 International Institute of Tropical Agriculture (IITA), Ibadan, Nigeria; 4 Department of Nematology, University of California Riverside, Riverside, CA, USA

**Keywords:** QTL analysis, Leaf morphology, Genomics, Genetics, Physical map, Synteny, Candidate genes, Cowpea, Legumes, EZA1/SWINGER

## Abstract

**Background:**

Cowpea [*Vigna unguiculata* (L.) Walp] exhibits a considerable variation in leaf shape. Although cowpea is mostly utilized as a dry grain and animal fodder crop, cowpea leaves are also used as a high-protein pot herb in many countries of Africa.

**Results:**

Leaf morphology was studied in the cowpea RIL population, Sanzi (sub-globose leaf shape) x Vita 7 (hastate leaf shape). A QTL for leaf shape, *Hls* (hastate leaf shape), was identified on the Sanzi x Vita 7 genetic map spanning from 56.54 cM to 67.54 cM distance on linkage group 15. SNP marker 1_0910 was the most significant over the two experiments, accounting for 74.7% phenotypic variance (LOD 33.82) in a greenhouse experiment and 71.5% phenotypic variance (LOD 30.89) in a field experiment. The corresponding *Hls* locus was positioned on the cowpea consensus genetic map on linkage group 4, spanning from 25.57 to 35.96 cM. A marker-trait association of the *Hls* region identified SNP marker 1_0349 alleles co-segregating with either the hastate or sub-globose leaf phenotype. High co-linearity was observed for the syntenic *Hls* region in *Medicago truncatula* and *Glycine max.* One syntenic locus for *Hls* was identified on Medicago chromosome 7 while syntenic regions for *Hls* were identified on two soybean chromosomes, 3 and 19. In all three syntenic loci, an ortholog for the EZA1/SWINGER (AT4G02020.1) gene was observed and is the candidate gene for the *Hls* locus. The *Hls* locus was identified on the cowpea physical map via SNP markers 1_0910, 1_1013 and 1_0992 which were identified in three BAC contigs; contig926, contig821 and contig25.

**Conclusions:**

This study has demonstrated how integrated genomic resources can be utilized for a candidate gene approach. Identification of genes which control leaf morphology may be utilized to improve the quality of cowpea leaves for vegetable and or forage markets as well as contribute to more fundamental research understanding the control of leaf shape in legumes.

## Background

Cowpea [*Vigna unguiculata* (L.) Walp] exhibits a considerable variation in leaf shape. Cowpea leaves are compound, having two asymmetrical side leaflets and one central terminal leaflet which is symmetrical. Typically, the central leaflet of the trifoliate is used in classifying the leaf shape due to variability of the side leaflets. In cowpea, the leaf shape is important for taxonomic classification and also for distinguishing cowpea varieties. However, there isn’t a central naming convention for cowpea leaves nor detailed descriptions of the leaf shapes, thus, many researchers name the leaf shapes differently. The two largest cowpea germplasm agencies are the International Institute of Tropical Agriculture (IITA) and the United States Department of Agriculture (USDA). IITA, which houses 14,500 cowpea accessions from 65 different countries, classifies cowpea leaf shapes into four categories, sub-globose, sub-hastate, globose and hastate/lanceolate (http://genebank.iita.org). The USDA, which houses 6,8411 cowpea accessions from 50 countries, classifies cowpea leaf shapes into five categories; globose, hastate, sub-globose, sub-hastate, strip and ovate-lanceolate (http://www.ars-grin.gov/cgi-bin/npgs/html/desclist.pl?188).

### Multipurpose cowpea

Cowpea is a multipurpose crop; the entire plant can be used for either human or livestock consumption. In 2009, cowpea dry grain production was estimated at 5,249,571 tons worldwide (http://faostat.fao.org). Although cowpea is not one of the highest production crops worldwide, nearly 90% of cowpea is produced in West Africa, which is estimated at 4,447,358 tons (http://faostat.fao.org). Cowpea is mainly grown in semi-arid regions by subsistence farmers, who sell the fresh or dried seeds, fresh pods and leaves as vegetables and the green or dried leftover parts of the plant, leaves and stems (haulms), can be used as fodder for livestock [1].

Young cowpea leaves are eaten as a pot herb and enjoyed in many parts of Africa. The freshly harvested leaves are sold in local markets in many parts of Ghana, Mali, Benin, Cameroon, Ethiopia, Uganda, Kenya, Tanzania and Malawi [2]. Cowpea shoots and leaves are rich sources of calcium, phosphorous and Vitamin B [3]. The young leaves are especially important in drought-prone regions of Sub-Saharan Africa to tide local populations over during the “hungry period” which occurs after planting but before the main harvest of fresh pods and dry grains. In Mozambique, dried cowpea seeds are mainly consumed by the poorer classes of people, whereas all social strata consume cowpea leaves eaten as a vegetable (personal communication, Rogerio Chiulele). Importantly, farmers can harvest and sell the young tender cowpea leaves while waiting for the cowpea grain crop to mature, which helps provide income to buy staple foods. Cowpea seedlings and tender young leaves are also a local delicacy and inherent to Zimbabwean cultures (personal communication, Wellington Muchero).

Dual purpose cowpea varieties which are bred for quality seeds, vegetables and fodder may add to a farmer’s revenue. For example, in Nigeria, farmers who sold dried cowpea fodder during the peak of the drought season saw a 25% increase to their annual income [4].

Although there is no emphasis in breeding cowpeas for the shape of their leaves, leaf shape is important for classifying and distinguishing cowpea varieties. The shape of the leaves may also be potentially useful as a morphological or physical marker used during the selection process if it is closely linked with an agronomic trait of interest. Interestingly, many wild cowpea relatives have the narrow or hastate leaf shape whereas most cultivated varieties of cowpea have the more common ovate or sub-globose leaf shape. However, any possible adaptive advantage for narrow leaves in wild cowpea has not been investigated. The hastate leaf shape was reported to be dominant to the ovate leaf shape in several studies [5-10]. This may indicate that the hastate shape is ancestral to the ovate leaf shape and the preponderance of the latter in most cultivated cowpea is due to direct or indirect selection by humans over time.

Molecular genetic tools and genomic resources have been developed for cowpea with an objective of enhancing breeding programs for the improvement of cowpea varieties for the United States, India, Brazil, and numerous countries in Africa and Asia. These integrated genomic resources include a 1536 SNP genotyping platform, an EST-derived SNP consensus genetic map, known syntenic relationships between cowpea, *Medicago truncatulaGlycine max* and *Arabidopsis thaliana*, and a cowpea EST sequence collection housed in HarvEST:Cowpea database (http://harvest.ucr.edu) [11,12]. A cowpea physical map has been partially anchored to the cowpea consensus genetic map using the same SNP markers (UCR cowpea group, unpublished) and is available publically (http://phymap.ucdavis.edu/cowpea). In addition, about 500 diverse cowpea accessions have been SNP-genotyped (UCR cowpea group, unpublished data) and a first draft of the cowpea genome, vs.0.02, has been assembled (http://www.harvest-blast.org). These resources will enable dissection of underlying genetic components of target agronomic traits using Quantitative Trait Locus (QTL) analysis and Association Mapping. The identified and confirmed QTLs will facilitate cultivar improvement using marker-assisted breeding.

In this study, we analyzed the genetics of leaf morphology in a segregating cowpea RIL population, Sanzi (sub-globose) x Vita7 (hastate). A QTL was identified for the “hastate leaf shape” locus, *Hls*, which was positioned on the cowpea consensus genetic map and cowpea physical map. A candidate gene was identified using syntenic relationships between cowpea, soybean and Medicago. In addition, a SNP marker was found which co-segregated with the leaf morphology genotypes and phenotype, which could be used as a molecular marker for breeding purposes. Future perspectives for this study are to fine map the *Hls* locus and identify cowpea candidate genes which would be utilized for more basic studies on leaf morphology in cowpea.

## Results and discussion

### Inheritance of leaf morphology

The inheritance of leaf morphology was studied using phenotypic data from one greenhouse experiment and one field experiment on the cowpea RIL population, Sanzi (sub-globose) x Vita 7 (hastate). The hastate and sub-globose leaf shape segregated 58:60 in the greenhouse experiment and 59:57 in the field experiment (*x*^2^_1:1_ = 0.03, p-value = 0.85) which fit the proposed model that the leaf shape is a qualitative trait (Table 1).

**Table 1:** Inheritance of leaf shape in Sanzi x Vita 7 population

**Experiment**	**Hastate**	**Sub-globose**	**Ratio**	***x***^**2**^	**p-value**
Greenhouse	58	60	1:1	0.03	0.85
Field	59	57	1:1	0.03	0.85

Several other researchers have studied the inheritance of the leaf shape in cowpea (hastate x ovate leaf shape) and reported that it was a qualitative trait [7,8,10,13]. Although the F_1_ generation was not assessed in the current study, the majority of researchers studying cowpea leaf shape have concluded that the hastate leaf shape is dominant to the more common ovate or sub-globose leaf shape [5-10]. However, Saunders et al. (1960b) reported that the hastate leaf shape was incompletely dominant to the ovate leaf shape.

### QTL analysis

**Figure 1:**
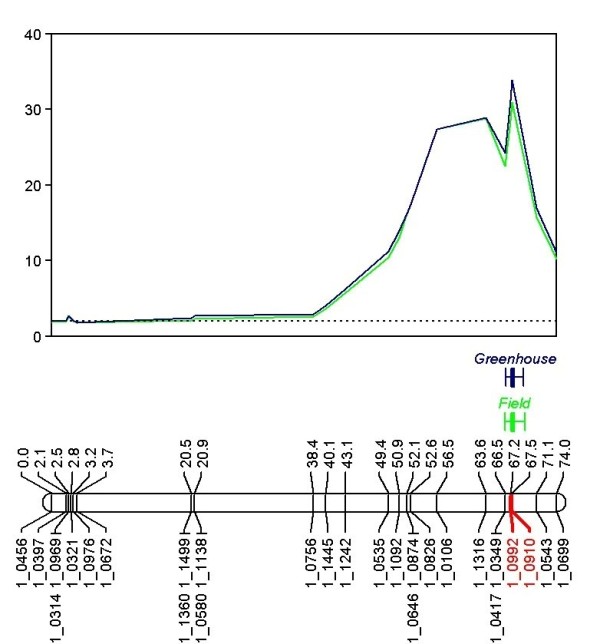
***Hls* locus on the Sanzi x Vita 7 genetic map.** Using Interval Mapping and Kruskal-Wallis analysis (only Interval Mapping analysis shown), *Hls* mapped to linkage group 15 on the Sanzi x Vita 7 genetic map, spanning from 56.54 cM to 67.54 cM. The greenhouse experiment data are plotted in blue and the field experiment data in green. SNP markers 1_0992 and 1_0910 are highlighted in red on the linkage group. The LOD significance threshold of 2.0 is indicated by a horizontal dotted line on the graph.

QTL analysis of the two phenotypic datasets identified one major QTL with a large effect for leaf shape morphology. The leaf morphology QTL spanned 11 cM distance on the Sanzi x Vita 7 individual genetic map from 56.54 cM to 67.54 cM on linkage group 15 (Figure 1, Tables 2, 3). SNP marker 1_0910 was the most significant marker in both of the datasets, accounting for 74.7% of the phenotypic variance (LOD 33.82) in the greenhouse experiment and 71.5% phenotypic variance (LOD 30.89) in the field experiment (Table  3). We propose the designation *Hls* (hastate leaf shape) for the QTL identified.

**Table 2:** **QTL analysis of the* Hls *locus in the Sanzi x Vita 7 population**

**Experiment**	**Analysis**	**1_0106**	**1_1316**	**1_0417**	**1_0349**	**1_0992**	**1_0910**
Greenhouse	IM LOD	27.32	28.8	24.18	24.18	31.21	33.82
	IM R^2^	66.2	69.1	62.7	62.7	71.9	74.7
	kw test statistic	76.12	78.68	71.38	71.38	81.74	84.91
	KW p-value	0.0001	0.0001	0.0001	0.0001	0.0001	0.0001
Field	IM LOD	27.29	28.77	22.44	22.44	28.57	30.89
	IM R^2^	66.2	69.1	59.9	59.9	68.7	71.5
	kw test statistic	76.08	78.62	68.30	68.30	78.15	81.31
	KW p-value	0.0001	0.0001	0.0001	0.0001	0.0001	0.0001

IM = Interval Mapping analysis, KW = Kruskal-Wallis analysis.

**Table 3:** **The* Hls *locus in the Sanzi x Vita 7 genetic map, cowpea consensus genetic map and cowpea physical map**

**Sanzi x Vita 7 genetic map**				**Cowpea consensus genetic map**			**Cowpea physical map**	
LG	cM	Locus	LOD	LG	cM	Locus	Contig	BAC clone(s)
15	56.55	1_0106	27.32	4	25.57	1_0106	383	CM056F01, CM067G06, CM007L11
		N/A		4	27.60	1_0678	1014	CH021P21
		N/A		4	27.90	1_1209	N/A	
		N/A		4	29.30	1_0117	N/A	
		N/A		4	29.51	1_0128	N/A	
15	63.65	1_1316	28.80	4	31.88	1_1316	N/A	
		N/A		4	32.21	1_0157	N/A	
		N/A		4	33.57	1_0038	926	CM002I07, CM052G13
		N/A		4	34.09	1_1013	926	CM050B03, CH004H23, CH046B08
15	67.54	1_0910	33.82	4	34.09	1_0910	821	CH050F07
15	67.20	1_0992	31.21	4	34.69	1_0992	25	CM041C03
		N/A		4	35.66	1_0083	N/A	
15	66.46	1_0349	24.18	4	35.87	1_0349	N/A	
15	66.46	1_0417	24.18	4	35.96	1_0417	N/A	

SNP markers are aligned in the order defined by the cowpea consensus genetic map.

Other researchers studying the inheritance of the hastate leaf shape in cowpea have reported a single dominant gene controlling the hastate leaf shape over the ovate or sub-globose leaf shape. Several gene symbols have been proposed, the first being *L*, which is a dominant gene controlling lanceolate leaf shape [14]. Ojomo et al. (1977) proposed the gene symbol *Ha* for the hastate leaf shape and Kolhe et al. (1970) proposed *Nlf* for narrow leaf shape. Fery (1980) proposed the gene symbol, *La,* for the narrow leaf shape. However, all of the studies investigating the narrow leaf shape used different cowpea accessions to make their populations. Whether these many studies are describing the same leaf shape locus or whether they are describing multiple independent loci remains inconclusive. Interestingly, Ogundiwin et al. (2005) identified one major QTL for the hastate leaf shape, designated *La*, in *Vigna unguiculata* ssp. *textilus*. Subspecies *textilus* is closely related to cultivated cowpea (*V. unguiculata* ssp. *unguiculata*); however, it does not easily hybridize. *La* could possibly be the syntenic locus of *Hls* in *V. textilus*.

The corresponding location of *Hls* was identified on the cowpea consensus genetic map. SNP markers which identified the *Hls* locus in the Sanzi x Vita 7 genetic map were aligned with the cowpea consensus genetic map (Table  3). The *Hls* locus spans from 25.57 cM to 35.96 cM on the cowpea consensus genetic map linkage group 4 (Table  3). The length of *Hls* on the individual genetic map, 11 cM, is nearly the same as on the cowpea consensus genetic map, 10.39 cM which may reflect accuracy of marker order (Table  3). The *Hls* locus on the cowpea consensus genetic map has several SNP markers which were not present in the Sanzi x Vita 7 population because of lack of polymorphism in the individual population (Table  3). In addition, there was a slight difference in the order of the SNP markers in the Sanzi x Vita7 population versus the cowpea consensus genetic map due to the merging of twelve individual genetic maps.

### Marker-trait association analysis

Seventeen diverse cowpea genotypes which have either the hastate or sub-globose leaf shape were used in a marker-trait association study to identify a SNP marker in the *Hls* region linked with the leaf shape phenotype. The hastate genotypes used for the analysis were selected from the USDA GRIN cowpea accession database and under their naming convention were classified as “strip” leaved. Vita 7, PI 632869, PI 632870, PI 632871, PI 632900, PI 632876, PI 632901, PI 632899 and PI 598341 were chosen for the hastate leaf shape phenotype (Additional file 1). PI 632882, CB27, Bambey 21, PI 418979, PI 448337 and PI 448682 were chosen from the USDA GRIN database and under their naming convention were classified as “sub-globose” leaf shape (Additional file 1). Accessions designated “TVNu” are wild cowpeas, many of which have the hastate leaf shape.

**Figure 2:**
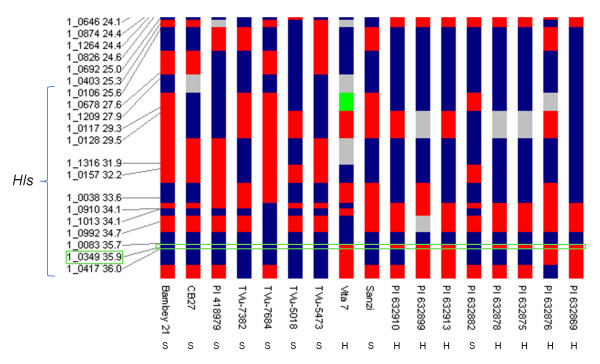
**Marker-trait association in the* Hls *locus.** The *Hls* locus on the cowpea consensus genetic map linkage group 4 is depicted vertically along with cowpea genotypes which differ in hastate or sub-globose leaf shape. Red colored blocks indicate the “AA” allele, blue colored blocks indicate the “BB” allele and grey colored blocks indicate that the locus has no detected SNP. Leaf shapes for cowpea accessions are labeled below: “S” indicates a sub-globose leaf shape and “H” indicates the hastate leaf shape. A marker-trait association was found for SNP marker 1_0349 (35.90 cM position) which is boxed in green. SNP marker 1_0349 co-segregated with the hastate and sub-globose leaf genotypes and the corresponding leaf phenotype. The allele for the hastate leaf genotype at this locus is the thymine nucleotide, color coded blue. The allele for the sub-globose genotype is the cytosine nucleotide, color coded red. The thymine/cytosine SNP for 1_0349 is at position 2122 in the cowpea P12 assembly unigene 8605 and can be viewed in HarvEST:Cowpea (http://harvest.ucr.edu).

The alleles of SNP marker 1_0349 (35.9 cM position) co-segregated perfectly with the hastate or sub-globose leaf phenotype (boxed in green in Figure 2). The allele for the hastate genotype at this locus was the thymine nucleotide (color coded blue in Figure 2). The allele for the sub-globose genotype was the cytosine nucleotide (color coded red in Figure 2). The thymine/cytosine SNP for 1_0349 is at position 2122 in the cowpea P12 assembly unigene 8605 and can be viewed in HarvEST:Cowpea (http://harvest.ucr.edu) (Additional file 2). The marker-trait association narrowed the *Hls* QTL to a 0.3 cM region and was defined by flanking SNP markers 1_0083 and 1_0417 (Figure 2).

### Candidate gene analysis using synteny with *M. truncatula* and *G. max*

The *Hls* locus was compared with the soybean, Medicago and Arabidopsis genomes to determine if a syntenic relationship exists. A high co-linearity or a conservation of gene order utilizing the EST-derived SNP markers with any of the sequenced genomes might reveal candidate genes. Synteny was examined using EST-derived SNP markers previously BLASTed and aligned to the soybean, Medicago and Arabidopsis genomes which are housed in the HarvEST:Cowpea database and are publicly available (http://harvest.ucr.edu). Due to limited resolution in the software images, not all markers are presented in the screenshot images output from Harvest:Cowpea. However, the cowpea consensus genetic map vs. 4 [12] has been used in fidelity. In order to view each individual marker, the linkage group must be magnified in the HarvEST:Cowpea database.

The *Hls* locus was examined for synteny with the Arabidopsis genome; however very low synteny was displayed at the macro level between cowpea and Arabidopsis so no further examination was pursued (Additional file 3).

**Figure 3:**
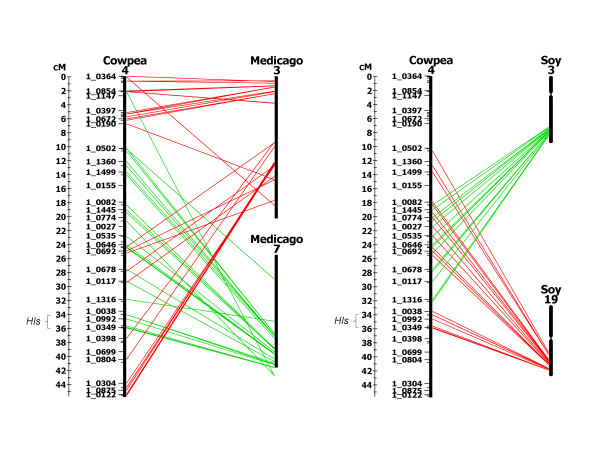
**Synteny of the *Hls* locus with *Medicago truncatula* and *Glycine max*.** Synteny was examined for the *Hls* locus between cowpea and *M. truncatula* and cowpea and *G. max* using EST-derived SNP markers previously BLASTed and aligned to the sequenced genomes. The *Hls* locus which spans 25.57 cM to 35.96 cM on linkage group 4 of the cowpea consensus genetic map was syntenic with Medicago chromosome 7. The syntenic locus spanned from Medicago locus Medtr7g084010 to Medtr7g134530. A candidate gene was identified in the highly significant syntenic region of *Hls*, Medtr7g133020, which was annotated as an ortholog of the Arabidopsis EZA1/SWINGER (SWN) gene. Two syntenic loci were identified for the *Hls* locus in soybean chromosomes 3 and 19. The syntenic region in soybean chromosome 3 spanned from the soybean locus Glyma03g34240 to Glyma03g38550. An orthologous candidate gene was observed in the most significant region of the syntenic *Hls* locus, Glyma03g38320, which was annotated as an ortholog of the Arabidopsis EZA1/SWINGER (SWN) gene. The syntenic *Hls* locus on soybean chromosome 19 spanned from Glyma19g36180 to Glyma19g41150 where another soybean ortholog of the EZA1/SWINGER (SWN) gene, Glyma19g40430, was observed. The syntenic map was drawn using HarvEST:Cowpea database (http://harvest.ucr.edu) using a cut off e-score value of −10 and a minimum number of 10 lines drawn per linkage group. Colored lines indicate cowpea genes orthologous to genes on *M. truncatula* and *G. max* chromosomes.

A high co-linearity was observed for the *Hls* locus with Medicago chromosome 7 (Figure 3, Table  4). Eight Medicago genes orthologous to cowpea SNP markers were identified in the syntenic region of Medicago chromosome 7 (Table  4). The syntenic region spanned from Medtr7g084010 locus to Medtr7g134530 locus which corresponded to 29.30 cM to 35.96 cM of the *Hls* locus on the cowpea consensus genetic map (Tables 3 and 4). The region which spanned from Medicago genes orthologous to cowpea SNP markers 1_1013 to 1_0349 were in the same linear order as on the cowpea consensus genetic map (Tables 3 and 4). The region spanning between Medicago genes orthologous to cowpea SNP markers 1_0910 (most significant marker in the QTL analysis) and 1_0349 (co-segregated with leaf genotype and phenotype) was examined for genes known to be associated with the molecular control of leaf morphology in other plant species [15] on the Medicago genome browser on the Phytozome webpage (http://www.phytozome.net). The Medicago locus Medtr7g133020 was observed between Medicago genes orthologous to cowpea SNP markers 1_0992 and 1_0083 and was annotated as an ortholog of the Arabidopsis gene AT4G02020.1 aka EZA1 or SWINGER (SWN) (Table  4). Medtr7g133020 has a SET domain (protein lysine methyltransferase enzyme) with two copies of a cysteine rich motif and is annotated as KOG: 1079; transcriptional repressor EZA1 (http://www.phytozome.net) (accessed April 2012).

**Table 4:** **The* Hls *syntenic region in* Medicago truncatula *chromosome 7**

**Medicago locus**	**Position (bp)**	**Phytozome annotation**	**Cowpea SNP**	**LG**	**cM**
Medtr7g084010	MtChr7: 18093097–18096342	Glycosyltransferase	1_1316	4	31.88
Medtr7g127710	MtChr7: 30002559–30004421	Small nuclear ribonucleoprotein G	1_0117	4	29.30
Medtr7g130340	MtChr7: 30448639–30451565	Tetrahydrofolate dehydrogenase/cyclohydrolase	1_1013	4	34.09
Medtr7g132610	MtChr7: 30739419–30778183	Histidine kinase	1_0910	4	34.09
Medtr7g132800	MtChr7: 30863955–30868447	Glycosyl hydrolase family 3 C terminal domain	1_0992	4	34.69
Medtr7g133020	MtChr7: 30974729–30981121	SWN (SWINGER); transcription factor	N/A	N/A	N/A
Medtr7g134340	MtChr7: 31708007–31710614	Peptidyl-prolyl cis-trans isomerase	1_0083	4	35.66
Medtr7g134420	MtChr7: 31747440–31752793	Papain family cysteine protease	1_0417	4	35.96
Medtr7g134530	MtChr7: 31793943–31799643	ATP-dependent RNA helicase	1_0349	4	35.87

The *Hls* region was examined for synteny with the soybean genome and was found to be highly co-linear with soybean chromosomes 3 and 19 (Figure 3, Table  5). Eight Medicago genes orthologous to cowpea SNP markers identified the region from locus Glyma03g34240 to Glyma03g38550 as the *Hls* syntenic locus in soybean chromosome 3 (Table  5). The soybean syntenic locus corresponded to 27.60 cM to 35.96 cM region in the *Hls* locus and was also in the same general marker order as the cowpea consensus genetic map (Table  5). The region spanning between orthologous soybean genes to cowpea SNP markers 1_1013 and 1_0349 was examined for leaf morphology candidate genes on the soybean genome browser on the Phytozome webpage (http://www.phytozome.net). Soybean locus Glyma03g38320 was observed flanked by orthologous genes for cowpea SNP markers 1_1013 and 1_0417 and was annotated as an ortholog of EZA1/SWINGER (SWN) gene. Glyma03g38320 has a SET domain (protein lysine methyltransferase enzyme) and two copies of a cysteine rich motif and is annotated as KOG: 1079; transcriptional repressor EZA1 (http://www.phytozome.net) (accessed April 2012).

**Table 5:** **The* Hls *syntenic region in* Glycine max *chromosomes 3 and 19**

***G. max chromosome***	***G. max*locus**	**Location (bp)**	**Phytozome annotation**	**Cowpea SNP**	**LG**	**cM**
3	Glyma03g34240	Gm03: 41726178–41732134	Protein phosphatase type 2A	1_1209	4	27.90 cM
3	Glyma03g34420	Gm03: 41865023–41866819	UDP glycosyl transferase	1_0678	4	27.60 cM
3	Glyma03g35490	Gm03: 42670842–42672212	Small nuclear ribonucleoprotein G	1_0117	4	29.30 cM
3	Glyma03g36050	Gm03: 43046482–43052190	Glycosyl transferase	1_1316	4	31.88 cM
3	Glyma03g36560	Gm03: 43503702–43504835	60S ribosomal protein	1_0157	4	32.21 cM
3	Glyma03g37080	Gm03: 43844395–43846689	Tetrahydrofolate dehydrogenase	1_1013	4	34.09 cM
3	Glyma03g38320	Gm03: 44664969–44672254	EZA1 (SWINGER); transcription factor	N/A	N/A	N/A
3	Glyma03g38520	Gm03: 44857426–44863787	Cysteine proteinase	1_0417	4	35.96 cM
3	Glyma03g38550	Gm03: 44884051–44889833	ATP-dependent RNA helicase	1_0349	4	35.87 cM
19	Glyma19g36180	Gm19: 43520883–43522581	60S ribosomal protein	1_0106	4	25.57 cM
19	Glyma19g36250	Gm19: 43594256–43596114	40S ribosomal protein S23	1_0061	2	24.10 cM
19	Glyma19g38130	Gm19: 45131688–45132559	Small nuclear ribonucleoprotein G	1_0117	4	29.30 cM
19	Glyma19g38170	Gm19: 45154806–45156026	Ubiquitin extension protein 2 (UBQ2)	1_0128	4	29.51 cM
19	Glyma19g38720	Gm19: 45583969–45589659	Glycosyl transferase	1_1316	4	31.88 cM
19	Glyma19g39170	Gm19: 45946131–45951841	Protein phosphatase	1_1349	3	39.80 cM
19	Glyma19g39240	Gm19: 45993099–45993972	60S ribosomal protein L21	1_0157	4	32.21 cM
19	Glyma19g39570	Gm19: 46201543–46203746	60S ribosomal protein L19	1_0038	4	33.57 cM
19	Glyma19g39710	Gm19: 46301684–46304736	Tetrahydrofolate dehydrogenase	1_1013	4	34.09 cM
19	Glyma19g40080	Gm19: 46544712–46546719	60S ribosomal protein L19	1_0038	4	33.57 cM
19	Glyma19g40090	Gm19: 46547961–46552179	Histidine kinase	1_0910	4	34.09 cM
19	Glyma19g40300	Gm19: 46736904–46743350	Glycosyl hydrolase family	1_0992	4	34.69 cM
19	Glyma19g40430	Gm19: 46838345–46844721	EZA1 (SWINGER); transcription factor	N/A	N/A	N/A
19	Glyma19g41120	Gm19: 47437575–47443343	Cysteine proteinase	1_0417	4	35.96 cM
19	Glyma19g41150	Gm19: 47465990–47471582	ATP-dependent RNA helicase	1_0349	4	35.87 cM

The *Hls* syntenic region in soybean chromosome 19 was identified by thirteen out of fourteen SNP markers, spanning from Glyma19g36180 to Glyma19g41150 which corresponded to 24.10 cM to 39.80 cM on the cowpea consensus genetic map (Table  5). The syntenic region in soybean between orthologous cowpea SNP markers 1_0910 and 1_0349 was examined for known leaf development genes using the soybean genome browser on the Phytozome webpage (http://www.phytozome.net). Glyma19g40430 locus was observed flanked by soybean genes orthologous to SNP markers 1_0992 and 1_0417 and was annotated as an ortholog of the Arabidopsis EZA1/SWINGER (SWN) gene (Table  5). Glyma19g40430 has a SET domain (protein lysine methyltransferase enzyme) and two copies of a cysteine rich motif and is annotated as KOG: 1079; transcriptional repressor EZA1 (http://www.phytozome.net) (accessed April 2012).

The candidate gene approach using syntenic relationships between cowpea, soybean and Medicago for the *Hls* locus identified orthologous candidate genes for the Arabidopsis gene AT4G02020.1 or EZA1/SWINGER (SWN). EZA1/SWINGER (SWN) is one of three Arabidopsis E(Z) orthologs of the *Drosophila melanogaster* gene ENHANCER OF ZESTE [E(Z)], which includes CURLY LEAF (CLF) and MEDEA (MEA) [16]. EZA1/SWINGER (SWN) is an H3K27 methyltransferase transcription factor and belongs to the Polycomb group proteins (Pc-G). Pc-Gs are involved in epigenetic regulation of developmental processes and are highly conserved in plants, animals and humans. In plants, Pc-G proteins are essential in regulating processes such as seed development [17], flower organ development [18-20] and leaf development [18,21].

CLF and SWN are expressed throughout many phases of plant development and have been shown to be involved in regulating leaf development. CLF is expressed during leaf and flower development [18] and EZA1/SWINGER is expressed in regions of dividing cells and meristems during vegetative and reproductive development [19]. CLF has been shown to directly target and repress the floral homeotic gene, AGAMOUS (AG), and a homeobox gene, SHOOTMERISTEMLESS (STM) [20,21]. SWN has been shown to have partially redundant functions with CLF and therefore may also be involved in regulating leaf development [19]. A *clf swn* double mutant produced narrow cotyledons, hypocotyls and roots and as it matured, the cotyledons developed finger-like growth on the margins as well as other abnormalities such as the shoot apex not developing leaves but a disorganized mass of undifferentiated tissue [19]. The fact that EZA1/SWINGER has been associated with leaf development in Arabidopsis makes it a plausible candidate gene for regulating leaf morphology in cowpea.

The combination of the marker-trait association and the identity of candidate genes in the syntenic loci enabled us to narrow the *Hls* region on the consensus genetic map, from 10.39 cM to approximately 1.87 cM distance. The narrowest distance between flanking markers to an orthologous candidate gene was in the Medicago locus, where Medtr7g133020 was flanked by SNP markers 1_0992 (34.69 cM position) and 1_0083 (35.66 cM position) which narrowed it to a 0.97 cM region. In soybean chromosome 19, the EZA1/SWINGER ortholog Glyma19g40430 was flanked by SNP markers 1_0992 (34.69 cM position) and 1_0417 (35.96 cM position) which narrowed the region to 1.37 cM. The furthest distance between flanking markers to orthologous candidate genes was in the syntenic locus in soybean chromosome 3, where Glyma03g38320 was flanked by SNP marker 1_1013 (34.09 cM position) and 1_0417 (35.96 cM position) with an approximate distance of 1.87 cM. On average, the most significant region in the *Hls* locus was narrowed to a 1.4 cM distance using the position of the candidate genes to narrow the QTL region. Assuming that the co-linearity of these three syntenous regions is upheld when extrapolated back to cowpea; the cowpea ortholog of EZA1/SWINGER should be present in this narrowed region.

Differences in marker significance under different analyses may be of interest. For example, SNP marker 1_0910 was the most significant in the QTL analysis while SNP marker 1_0349 co-segregated with the genotype and phenotype for leaf shape. QTL analysis often identifies large confidence intervals depending on the heritability of the trait and because all genes on a chromosome will show some linkage amongst themselves, a QTL will be associated with several markers [22]. This was the case for SNP markers 1_0349 and 1_0910, which are 1.08 cM distance apart on the individual genetic map and 1.78 cM on the cowpea consensus genetic map (Table  3). We have found that small phenotyping differences between experiments may move the most significant marker by 1 cM or more. The marker-trait association in which SNP marker 1_0349 co-segregated with the genotype and phenotype for leaf shape utilized a simplified haplotype analysis, where unrelated individuals were examined for inheritance of alleles within a specific region. The synteny study revealed that Medicago and soybean orthologs to cowpea SNP markers 1_0083, 1_0092, 1_1013 and 1_0417 were flanking the EZA1 candidate genes (Tables 4 and 5, Additional file 4). These four markers flank the most significant marker from the QTL analysis, 1_0910, and 1_0349 which co-segregated with the genotype and phenotype for leaf shape (Additional file 4). By utilizing QTL analysis, marker-trait association and candidate gene analysis using synteny, validation was provided that the genetic determinant is most likely located within a 1.37 cM region of closely linked markers.

### Leaf morphology candidate genes BLAST to cowpea genomic resources

The genomic sequences for Medtr7g133020, Glyma03g38320, Glyma19g40430 and the Arabidopsis EZA1 gene (AT4G02020.1) were BLASTed to the cowpea genome vs. 02 (http://www.harvest-blast.org) and HarvEST:Cowpea database (http://harvest.ucr.edu) to identify orthologous cowpea sequences. The Medtr7g133020 and AT4G02020.1 genomic sequences returned a high BLAST alignment with contig C27495629 (Table  6). The genomic sequences for Glyma03g38320 and Glyma19g40430 returned a high alignment with contig C27664167 and scaffold28398 (Table 6). All genomic sequences when BLASTed to Harvest:Cowpea database returned the best alignment with cowpea unigene 21752 which was annotated as an EZA1 ortholog (Table  6). Interestingly, unigene 21752 was obtained from leaf and shoot meristems used for a mature pre-flowering developmental stage cDNA library from cowpea varieties DanIla, Tvu11986, Tvu7778 and 12008D (http://harvest.ucr.edu). The genomic and unigene sequences identified for the cowpea ortholog for EZA1 will enable future studies to clone and confirm the candidate gene.

**Table 6:** Medicago, soybean and Arabidopsis EZA1/SWINGER genes BLAST to cowpea genomic resources

**EZA1(SWINGER) ortholog**	**Cowpea genome**	**e-score**	**Cowpea unigene**	**e-score**
Medtr7g133020	C27495629	1.00E-15	21752	4.00E-11
Glyma03g38320	C27664167	7.00E-30	21752	1.00E-17
Glyma19g40430	scaffold28398	6.00E-36	21752	6.00E-10
AT4G02020.1	C27495629	3.00E-22	21752	9.00E-21

### *Hls* in the cowpea physical map

The cowpea physical map (http://phymap.ucdavis.edu/cowpea) which has been partially anchored to the cowpea consensus genetic map via the same SNP markers was used to identify BAC contigs which span the *Hls* region.

Significant markers from the QTL study and closely linked markers from the cowpea consensus genetic map identified several BAC contigs which incompletely span the *Hls* region (Table  3). The most significant SNP marker from the QTL analysis, 1_0910, was identified in BAC clone CH050F07 of contig821 (Table  3). Contig821 has four overlapping BAC clones and 128 non-repeating bands which estimated the contig size at 209,920 bp (http://phymap.ucdavis.edu/cowpea). SNP marker 1_0992 which was closely linked with the EZA1 candidate gene in two out of three of the syntenic loci, was identified in BAC clone CM041C03 of contig25 (Table  3). Contig25 has 731 overlapping BAC clones and 1843 non-repeated bands which estimated the length as 3,022,520 bp (http://phymap.ucdavis.edu/cowpea) (Table  3). The combined length of the two BAC contigs which span the most significant region of the *Hls* QTL is 3,232,440 bp. Since SNP marker 1_0992 was closely linked to the EZA1/SWINGER candidate gene in the *Hls* syntenic locus in Medicago chromosome 7 and soybean chromosome 19, the cowpea EZA1 gene may be located on BAC contig25. Currently, there are BAC-end sequences (BES) of approximately 700 bp for clones in the minimum tiling path (MTP) of BAC contigs in the cowpea physical map. However, none of the BESs of clones in either contig25 or contig821 yielded cowpea EZA1 genes when BLASTed to the HarvEST:Cowpea database. Future perspectives for enhancing the cowpea physical map may include sequencing BAC clones within the MTP of each BAC contig which would enable the direct identification of genes of interest.

To test the candidacy of the cowpea EZA1 gene for the *Hls* locus, a molecular marker could be developed and mapped to ensure it co-locates in the *Hls* locus in the Sanzi x Vita 7 population. Additionally, the cowpea EZA1 gene would need to be cloned and sequenced from both parents to determine the allelic variation for phenotype followed by complementation tests to validate gene function.

## Conclusion

This study has identified one major QTL, *Hls*, which is associated with the hastate and sub-globose leaf shape in the cowpea RIL population Sanzi x Vita 7. Our candidate gene approach utilized mapping the locus and a marker-trait association to narrow the QTL locus of 11 cM to one marker which co-segregated with the trait. The conserved gene order amongst closely related species, cowpea and soybean, and members within the same legume family, cowpea, Medicago and soybean, enabled the identification of a candidate gene for the *Hls* locus. Future goals will be to utilize the molecular marker which co-segregated with leaf shape in MAS breeding efforts. A more fundamental study could also be undertaken to determine if the candidate gene EZA1/SWINGER is the genetic determinant governing leaf morphology in cowpea.

## Methods

### Plant population

Leaf morphology was studied in a cowpea RIL population which was developed from an intraspecific cross of Sanzi x Vita 7. The population consisted of 122 RILs which were advanced by single seed descent to the F_10_ generation. Sanzi is a local landrace from Ghana which has a prostrate sprawling architecture, grayish-purple seeds, and a sub-globose leaf shape. Vita 7 (PI 580806/TVu-8461) is an IITA advanced breeding line from Nigeria with an upright bush type architecture, beige seeds and hastate leaf shape (IITA germplasm database online; http://genebank.iita.org). The Sanzi x Vita 7 population was received from Christian Fatokun, IITA, Ibadan, Nigeria. All cowpea accessions were available from the University of California Riverside cowpea germplasm collection.

### Phenotyping

**Figure 4:**
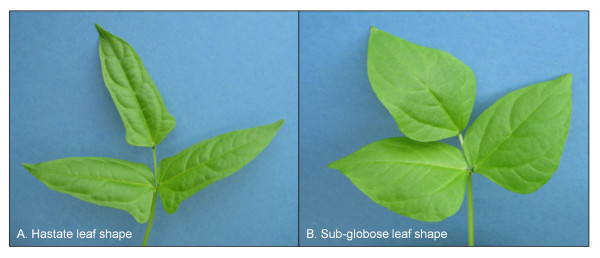
Hastate and sub-globose leaf shapes segregating in the Sanzi x Vita7 population.

The terminal central leaflet was observed and classified as “hastate” or “sub-globose” (Figure 4) five weeks after germination for each of the RILs. Two sets of phenotypic data were obtained; one dataset during a greenhouse experiment and the second dataset during a field experiment. The greenhouse study, which phenotyped 118 out of 122 RILs, was conducted from February to April 2010 in Riverside, California. Seedlings were transplanted into 3785 cm^3^ pots and watered daily, with day and night temperatures set to 28°C and 16°C, respectively. The field experiment, which phenotyped 116 out of 122 RILs, was conducted at the Citrus Research Center-Agricultural Experiment Station (CRC-AES) in Riverside CA, from July to September 2010. Twenty-five seeds per replicate were planted for each RIL in a randomized complete block design using four replicates. Seeds were machine-planted in single rows on pre-irrigated raised beds spaced 76 cm apart with 10 cm spacing between seeds.

### SNP genotyping

The Sanzi x Vita 7 population was genotyped at the F_8_ generation using bi-allelic SNP markers from the 1536 Illumina GoldenGate Assay as previously described [11]. All genotypes used for the marker-trait association study were SNP genotyped at the F_8_ generation or above as previously described [11].

### Genetic map

A SNP genetic map was developed previously for the Sanzi x Vita 7 RIL population and is included in the cowpea consensus genetic map vs.4 [12]. The individual map was generated using 122 RILs and 416 SNP markers. The map consists of nineteen linkage groups and spans approximately 753 cM total distance.

### Cowpea consensus genetic map

The cowpea consensus genetic map vs. 4, which is an updated version of the Muchero et al. 2009 map, was used for this study [12]. The consensus version 4 map consists of ten RIL populations and two F_4_ breeding populations, which has increased the marker density and improved the marker order. The map is 680 cM in length and contains 1107 markers with an average of 0.65 cM between markers. The current SNP-based cowpea linkage map is included in a publicly available browser called HarvEST:Cowpea, which can be downloaded from http://harvest.ucr.edu or viewed online at http://www.harvest-web.org.

### Statistical analysis

The Kruskal-Wallis and Interval Mapping analysis packages of MapQTL 5.0 software were used to conduct the QTL analysis [23]. A QTL was considered significant if the same QTL was identified using both phenotypic datasets and if the statistical tests for the markers met significance thresholds for both Kruskal-Wallis and Interval Mapping analyses. A significance threshold was set to 0.05 for Kruskal-Wallis analysis and LOD thresholds for the Interval Mapping analysis were calculated using 1000 permutations at the 0.05 significance level. A 95% confidence interval was used to estimate the left and right margins of the QTL using 1-LOD and 2-LOD of the most likely position. QTLs were visualized using MapChart 2.2 software [24].

### Synteny

Synteny was examined for cowpea with *G. max*, *M. truncatula* and *A. thaliana* using EST-derived SNP markers previously BLASTed and aligned to the sequenced genomes. Annotations for the soybean and Medicago loci were taken directly from the Phytozome website (http://www.phytozome.org). Syntenic relationships amongst the different genomes can be examined in the HarvEST:Cowpea database (http://harvest.ucr.edu). Syntenic maps were drawn using HarvEST:Cowpea using a cut-off e-score value of -10, with a minimum number of 10 lines drawn per linkage group.

### Marker-trait association

Genotypic data comprised of cowpea varieties and SNP marker information in the *Hls* locus were visualized using GGT 2.0 software [25]. The cowpea consensus genetic map vs.4 [12] was loaded into the software to visualize linkage groups.

### Cowpea physical map

The physical map was developed using an advanced African breeding line IT97K-499-35 (http://phymap.ucdavis.edu/cowpea). It consists of two BAC clone libraries developed using restriction enzymes *Hind*III and *Mbo*I (Amplicon Express, Pullman, WA). Contigs were assembled using the snapshot method of DNA fingerprinting [26] and completed at University of California Davis by Ming Cheng Luo. The final physical map is an assembly of 43,717 BACs with an 11x genome depth of coverage. The size of the BAC clones was estimated by multiplying the number of unique bands generated from the fingerprinting assay by 1640 bp (personal communication, Ming Cheng Luo).

## Abbreviations

BAC, Bacterial artificial chromosome; BES, BAC end sequence; bp, Base pairs; cM, Centimorgans; EST, Expressed sequence tags; EZA1, ENHANCER OF ZESTE; LG, Linkage group; LOD, Logarithm of (base 10) of odds; MAS, Marker-assisted selection; Mb, Megabases; MTP, Minimum tiling path; Pc-G, Polycomb-group protein; QTL, Quantitative trait locus; RIL, Recombinant inbred line; SNPs, Single nucleotide polymorphism; SWN, SWINGER.

## Competing interests

The author(s) declare that they have no competing interests.

## Authors’ contributions

MP conducted the greenhouse and field experiments. MP analyzed the genetic inheritance, QTL analysis, marker-trait association, candidate gene analysis using synteny and comparison of the cowpea consensus genetic map and physical map. CF provided the RIL population. MP, JDE, PAR and TJC participated in the design, interpretation of data and writing of the manuscript. All authors read and approved the final manuscript.

## Funding declaration

This work was supported in part by the Generation Challenge Program through a grant from the Bill and Melinda Gates Foundation, the U.S. Agency for International Development Collaborative Research Support Program Grants GDG-G-00-02-00012-00 and EDH-A-00-07-00005 and the University of California Agricultural Experiment Station.

## Supplementary Material

Additional file 1 Cowpea accessions with a hastate or sub-globose leaf phenotype. Click here for file

Additional file 2 **SNP marker 1_0349 sequence.** cDNA sequence of P12 assembly unigene 8605 which is housed in Harvest:Cowpea database (http://harvest.ucr.edu). The SNP (thymine/cytosine) is located at position 2122, parenthesized, underlined and in bold. Click here for file

Additional file 3 **Synteny of the Hls locus with A. *thaliana*.** Synteny was examined for the *Hls* locus between cowpea and A. *thaliana* using EST-derived SNP markers previously BLASTed and aligned to the sequenced genome. The *Hls* locus on the cowpea consensus genetic map, linkage group 4 (25.57 cM – 35.96 cM position), showed very low synteny with the Arabidopsis genome. The syntenic map was drawn using HarvEST:Cowpea database (http://harvest.ucr.edu) using a cut off e-score value of -10 and a minimum number of 10 lines drawn per linkage group. Click here for file

Additional file 4 **Summary of significant markers in the* Hls *locus.** Click here for file

## References

[B1] Inaizumi H Singh BB Sanginga PC Manyong VM Adesina AA Tarawali S Adoption and Impact of Dry-season Dual-purpose Cowpea in the Semiarid Zone of Nigeria 1999 International Institute of Tropical Agriculture (IITA), Ibadan

[B2] Barrett RP Integrating Leaf and Seed Production Strategies for Cowpea (Vigna unguiculata (L.) Walp.) 1987 Michigan State University, East Lansing

[B3] Maynard DN Underutilized and underexploited horticultural crops Hortscience 2008 43 279

[B4] Dugje IY Omoigui LO Ekeleme F Kamara AY Ajeigbe H Farmers’ Guide to Cowpea Production in West Africa 2009 International Institute of Tropical Agriculture (IITA), Ibadan

[B5] Krishnaswamy N Nambiar KK Mariakulandai A Studies on cowpea [Vigna unguiculata (L.) Walp.] Madras Agric J 1945 33 145 160

[B6] Jindla L Singh BB Inheritance of flower color leaf shape and pod length in cowpea (Vigna sinensis L.) Indian J Hered 1970 2 45 49

[B7] Ojomo OA Morphology and genetics of two gene markers, ‘Swollen stem base’ and ‘Hastate leaf’ in cowpea, Vigna unguiculata (L) Walp J Agric Sci 1977 88 227 231 10.1017/S0021859600033992

[B8] Kohle AK Genetic studies in Vigna sp Poona Agric Coll Mag 1970 59 126 137

[B9] Fery RL Singh SR, Rachie KO The genetics of cowpea: a review of the world literature Cowpea Research, Production and Utilization 1985 John Wiley and Sons, Chichester 25 62

[B10] Oluwatosin OB Inheritance of genes for leaflet shape and leaflet shape modifier in cowpea Afr Crop Sci J 2002 10 133 137

[B11] Muchero W Diop NN Bhat PR Fenton RD Wanamaker S Pottorff M Hearne S Cisse N Fatokun C Ehlers JD A consensus genetic map of cowpea [Vigna unguiculata (L) Walp.] and synteny based on EST-derived SNPs Proc Natl Acad Sci 2009 106 18159 18164 10.1073/pnas.0905886106 19826088 PMC2761239

[B12] Lucas MR Diop NN Wanamaker S Ehlers JD Roberts PA Close TJ Cowpea–soybean synteny clarified through an improved genetic map Plant Genome J 2011 4 218 225 10.3835/plantgenome2011.06.0019

[B13] Saunders AR Inheritance in the cowpea III: mutations and linkages S Afr J Agric Sci 1960 3 327 348

[B14] Harland SC Inheritance of certain characters in the cowpea (Vigna sinensis) J Genet 1919 8 101 132 10.1007/BF02983490

[B15] Barkoulas M Galinha C Grigg SP Tsiantis M From genes to shape: regulatory interactions in leaf development Curr Opin Plant Biol 2007 10 660 666 10.1016/j.pbi.2007.07.012 17869569

[B16] Guitton A Berger F Control of reproduction by Polycomb Group complexes in animals and plants Int J Dev Biol 2005 49 707 716 10.1387/ijdb.051990ag 16096976

[B17] Wang D Tyson MD Jackson SS Yadegari R Partially redundant functions of two SET-domain polycomb-group proteins in controlling initiation of seed development in Arabidopsis Proc Natl Acad Sci 2006 103 13244 13249 10.1073/pnas.0605551103 16924116 PMC1559784

[B18] Goodrich J Puangsomlee P Martin M Long D Meyerowitz EM Coupland G A polycomb-group gene regulates homeotic gene expression in Arabidopsis Nature 1997 386 44 51 10.1038/386044a0 9052779

[B19] Chanvivattana Y Bishopp A Schubert D Stock C Moon YH Sung ZR Goodrich J Interaction of polycomb-group proteins controlling flowering in Arabidopsis Development 2004 131 5263 5276 10.1242/dev.01400 15456723

[B20] Schubert D Primavesi L Bishopp A Roberts G Doonan J Jenuwein T Goodrich J Silencing by plant polycomb-group genes requires dispersed trimethylation of histone H3 at lysine 27 EMBO J 2006 25 4638 4649 10.1038/sj.emboj.7601311 16957776 PMC1590001

[B21] Katz A Oliva M Mosquna A Hakim O Ohad N FIE and CURLY LEAF polycomb proteins interact in the regulation of homeobox gene expression during sporophyte development Plant J 2004 37 707 719 10.1111/j.1365-313X.2003.01996.x 14871310

[B22] Kearsey MJ Farquhar AGL QTL analysis in plants; where are we now? Heredity 1998 80 137 142 10.1046/j.1365-2540.1998.00500.x 9503632

[B23] Van Ooijen JW MapQTL® 5, Software for the Mapping of Quantitative Trait Loci in Experimental Populations 2004 Kyazma BV, Wageningen

[B24] Voorrips RE MapChart: software for the graphical presentation of linkage maps and QTLs J Hered 2002 93 77 78 10.1093/jhered/93.1.77 12011185

[B25] Van Berloo R GGT 2.0: versatile software for visualization and analysis of genetic data J Hered 2008 99 232 236 10.1093/jhered/esm109 18222930

[B26] Luo MC Thomas C You FM Hsiao J Ouyang S Buell CR Malandro M McGuire PE Anderson OD Dvorak J High-throughput fingerprinting of bacterial artificial chromosomes using the snapshot labeling kit and sizing of restriction fragments by capillary electrophoresis Genomics 2003 82 378 389 10.1016/S0888-7543(03)00128-9 12906862

