# Supplementary material for: Leaf morphology in Cowpea [Vigna unguiculata (L.) Walp]: QTL analysis, physical mapping and identifying a candidate gene using synteny with model legume species
Source: BMC Genomics. 2012 Jun 12;13:234. doi: 10.1186/1471-2164-13-234 (PMC3431217; doi:10.1186/1471-2164-13-234)
Supplement: Additional file 1 — Cowpea accessions with a hastate or sub-globose leaf phenotype. [file 1471-2164-13-234-S1.docx]

| Additional file 1. Cowpea accessions with a hastate or sub-globose leaf phenotype. | | | |
| --- | --- | --- | --- |
| Hastate or "strip" leaf shape | Source/Origin | Sub-globose leaf shape | Source/Origin |
| Vita7/ PI 580806/ TVu 8461 | IITA/Nigeria | Sanzi | Ghana |
| PI 632869/ TVNu 435 | Malawi | California Blackeye 27 (CB27) | United States |
| PI 632875/ TVNu 523 | Zambia | Bambey21 | Senegal |
| PI 632876/ TVNu 531 | Tanzania | PI 418979/ HAN CHUI YEN | Shaanxi, China |
| PI 632878/ TVNu 554 | Zambia | PI 448337/ TVu 5018 | Niger |
| PI 632899/ TVNu 113 | Tanzania | PI 448682/ TVu 5473 | Niger |
| PI 632910/ TVNu 109 | Tanzania | PI 580445/ TVu 7382/ UCR 4734 | Nigeria |
| PI 632913/ TVNu 353 | Zambia | PI 580510/ TVu 7684/ UCR 4785 | Nigeria |
|  |  | PI 632882/ TVNu 671 | Niger |
