# Supplementary material for: Leaf morphology in Cowpea [Vigna unguiculata (L.) Walp]: QTL analysis, physical mapping and identifying a candidate gene using synteny with model legume species
Source: BMC Genomics. 2012 Jun 12;13:234. doi: 10.1186/1471-2164-13-234 (PMC3431217; doi:10.1186/1471-2164-13-234)
Supplement: Additional file 2 — SNP marker 1_0349 sequence. cDNA sequence of P12 assembly unigene 8605 which is housed in Harvest:Cowpea database (http://harvest.ucr.edu). The SNP (thymine/cytosine) is located at position 2122, parenthesized, underlined and in bold. [file 1471-2164-13-234-S2.docx]

CATTGCTCGCGCGAAGACCGGTACTGGAAAGACGCTAGCGTTCGGAATTCCAGTTATTAAAGGCCTCACTGAAGTTGAAGATGAGCCTTCTCTCAGGAGGTCTGGTAGGCTTCCCAGAGTTTTGGTGCTGGCCCCTACGAGGGAGTTGGCGAAGCAAGTGGAGAAGGAGATAAAGGAATCTGCTCCTTATCTCAGCACTGTTTGTGTTTATGGCGGTGTTTCTTATGTTACTCAGCAGAGTGCTCTTTCACGAGGTGTAGATGTGGTGGTCGGGACCCCAGGGAGAATAATTGACTTGATTAATGGGAAGAGCCTTAAGCTGAATGAGGTTCAGTATTTGGTGCTTGATGAAGCAGATCAGATGCTTGCTGTTGGGTTTGAGGAGGATGTGGAAGTGATTTTAGAGAACCTCCCTTCTCAGAGGCAGAGCATGCTTTTCTCTGCCACCATGCCTGCTTGGGTGAAGAAGTTGGCGAGAAAATATTTGAACAACCCACTCACAATTGATTTGGTTGGTGATGAAGAAGAAAAGCTCGCTGAAGGGATAAAACTTTTTGCTATATCAGCCACTGCCACTTCAAAGCGGACAATTCTCTCTGATCTCGTAACTGTTTATGCAAAGGGTGGGAAGACTATTGTATTTACACAGACAAAAAAAGATGCTGATGAAGTATCACTGTCATTAACAAATAGTATAACGTCTGAAGCACTGCATGGTGATATATCTCAGCATCAGAGAGAAAGAACATTGAATGGTTTTCGGCAAGGAAAATTCACAGTGCTTGTTGCTACTGATGTTGCAGCTCGTGGACTTGATATTCCCAATGTTGATTTGATTATCCATTATGAGCTTCCCAATGATCCTGAGACTTTCGTACACCGCTCTGGTCGTACTGGTCGTGCTGGAAAACAAGGTACTGCCATTCTGTTGTACACCAGTAGCCAGAGGAGAACAGTTAGATCCCTTGAACGTGATGTAGGCTGCAAGTTTGAATTTGTTAGTCCGCCAGCTATGGAAGAGGTCTTGGAGTCATCTGCGGAGCAGGTTGTTGCCACACTTGGTGGAGTTCATCCCGAATCTATCCAGTTTTTCACCCCAACTGCACAAAAACTGATCGAAGAACAAGGAACAACTGCCCTTGCCGCTGCCCTTGCACAACTTAGTGGATTTTCCCGACCTCCATCATCCCGGTCTCTTATCACCCACGAACAGGGATGGACTACGTTGCAACTAATTCGGGATTCGGAGAATAGTAGATATTTTTCAGCAAGATCAGTCACTGGGTTTCTTTCTGATGTTTTTTCATCAGCTGCCGATGAAGTTGGAAAAATCCATATAATTGCAGATGAAAGGGTTCAAGGAGCCGTTTTTGATCTTCCCGAGGAGATTGCTAAAGAGTTGCTTACTAAGGACATACCACCTGGTAACACCATTTCCAAGATCACCAAGCTACCTCCTTTGCAAGACGATGGGCCTCCAAGTGATTTCTATGGAAGGTTCTCTGACAGAGAACGTGGTAACCGAAGAGGATCTACTTCTAGGGGAGGTTTTAGTTCTAGGGGAGGTGGTTTTGCTTCTAGGGACCGGAGAGGTTTTAAATCCTCACGGGGATGGGATGGGGAAGACTCTGATGATGACGACTTCAGTGATCGATCTAGTAGGAGAGGTGGTAGAAATTTTAAATCTGGCGGCAATAGCTGGTCTCGAGCAGGAGGTAAAAGTGGTGGAGATGATTGGCTAATTGGGGGTAGACGATCAAGCCGGCCTTCATCATCAGACAGATTCGGAGGGGCCTGTTTCAATTGTGGGGAATCTGGTCATCGTGCATCAGATTGTCCAAACTCTTCAAACCGGCGAAGCTTTTTTTAAGTTCCCACATTTTTTTGGGGCGCCGCTTTGACCATGACGGACATGAACTTGTGCCACTGTTATTGGCCTGATGGGTTCCGGAAAATTGAAGCATGCTTACCGAAAAGAGTTACAGAAGCAATATTAGTTTGCATCTCACGTGTTGGCGTGATCTCCGTGGGGACCTCCTTTGTCGTCCTCTTTTTTGTGTCTCAATGAAATTTAGTATTTGTTTGGGCTTAAGAATAGTGCTGTATCTTCTTTTTCGGGTT**(C/T)**GGTTTAAGAGGTTAGTTGTATGGTCCTGTATTCTTCTCAACTTATTATTTAACATCTTTTTGAACCTTCCCGGTTTAGGAACAGACTGGAAAAATGAATGAAAGATGAAATCCTAAAGGTTTATGCAAAAAAAAAAAAAAAAAA
