# Supplementary figures and images for: Leaf morphology in Cowpea [Vigna unguiculata (L.) Walp]: QTL analysis, physical mapping and identifying a candidate gene using synteny with model legume species
Source: BMC Genomics. 2012 Jun 12;13:234. doi: 10.1186/1471-2164-13-234 (PMC3431217; doi:10.1186/1471-2164-13-234)

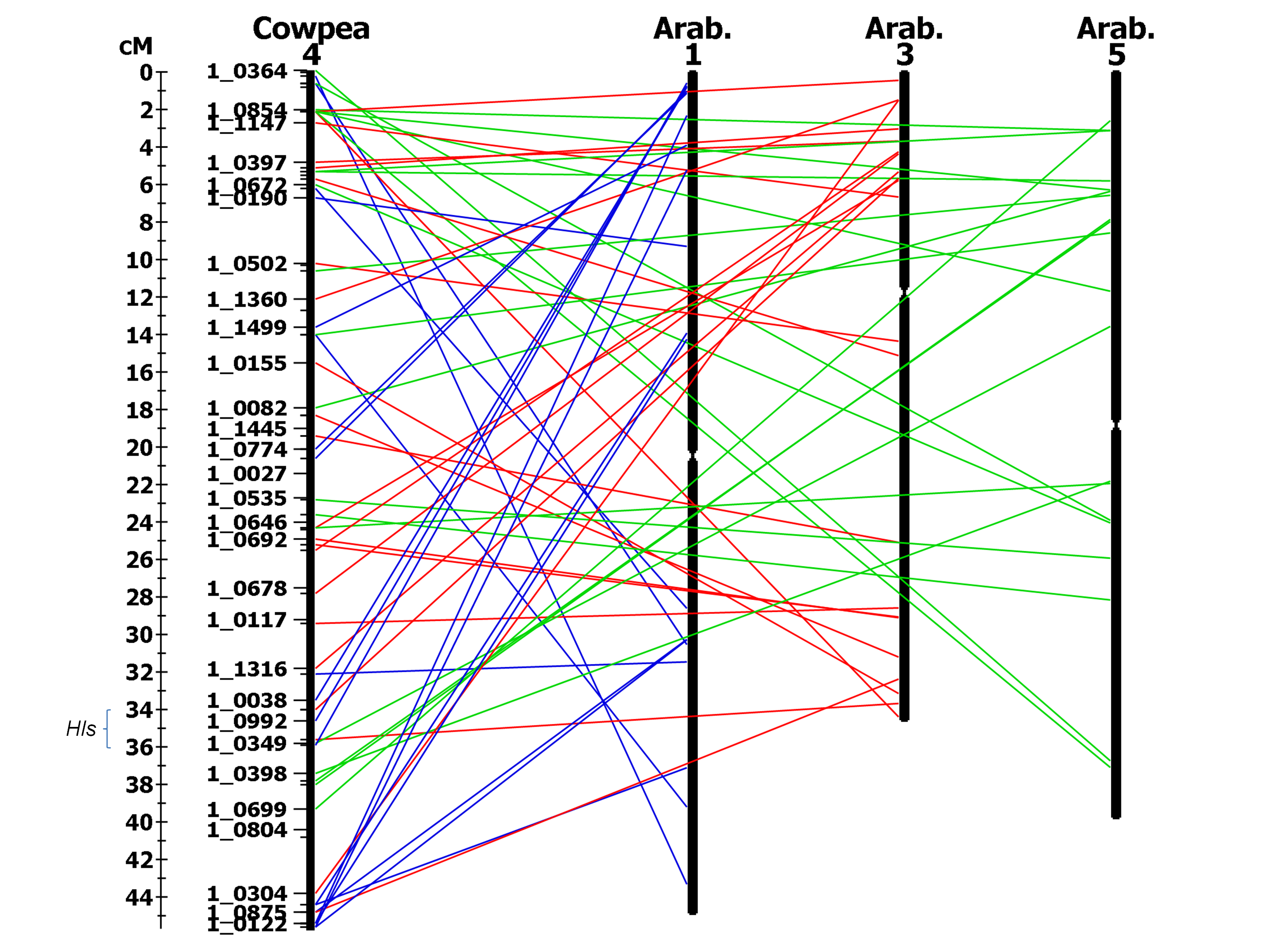

Supplement: Additional file 3 — Synteny of the Hls locus with A. thaliana. Synteny was examined for the Hls locus between cowpea and A. thaliana using EST-derived SNP markers previously BLASTed and aligned to the sequenced genome. The Hls locus on the cowpea consensus genetic map, linkage group 4 (25.57 cM – 35.96 cM position), showed very low synteny with the Arabidopsis genome. The syntenic map was drawn using HarvEST:Cowpea database (http://harvest.ucr.edu) using a cut off e-score value of -10 and a minimum number of 10 lines drawn per linkage group. [file 1471-2164-13-234-S3.tiff]
