# Supplementary material for: Leaf morphology in Cowpea [Vigna unguiculata (L.) Walp]: QTL analysis, physical mapping and identifying a candidate gene using synteny with model legume species
Source: BMC Genomics. 2012 Jun 12;13:234. doi: 10.1186/1471-2164-13-234 (PMC3431217; doi:10.1186/1471-2164-13-234)
Supplement: Additional file 4 — Summary of significant markers in the Hls locus. [file 1471-2164-13-234-S4.docx]

| Additional file 4. Summary of significant markers in the *Hls* locus. | | | | | | | |
| --- | --- | --- | --- | --- | --- | --- | --- |
| Analysis | Description | Sanzi x Vita 7 | | | Cowpea genetic map | | |
|  |  | SNP | LG | cM | SNP | LG | cM |
| Synteny | Flanking marker to candidate gene |  |  |  | 1_1013 | 4 | 34.09 |
| QTL analysis | Most significant marker in QTL analysis | 1_0910 | 15 | 67.54 | 1_0910 | 4 | 34.09 |
| Synteny | Flanking marker to candidate gene | 1_0992 | 15 | 67.20 | 1_0992 | 4 | 34.69 |
| Synteny | Flanking marker to candidate gene |  |  |  | 1_0083 | 4 | 35.66 |
| Marker-trait association | Co-segregated with genotype/phenotype | 1_0349 | 15 | 66.46 | 1_0349 | 4 | 35.87 |
| Synteny | Flanking marker to candidate gene | 1_0417 | 15 | 66.46 | 1_0417 | 4 | 35.96 |

SNP markers are aligned to the order defined by the cowpea consensus genetic map.
